# Route of inoculation and mosquito vector exposure modulate dengue virus replication kinetics and immune responses in rhesus macaques

**DOI:** 10.1371/journal.pntd.0008191

**Published:** 2020-04-08

**Authors:** Michael K. McCracken, Gregory D. Gromowski, Lindsey S. Garver, Brad A. Goupil, Kathryne D. Walker, Heather Friberg, Jeffrey R. Currier, Wiriya Rutvisuttinunt, Kevin L. Hinton, Rebecca C. Christofferson, Christopher N. Mores, Yannick Vanloubbeeck, Clarisse Lorin, Marie-Pierre Malice, Stephen J. Thomas, Richard G. Jarman, David W. Vaughn, J. Robert Putnak, Lucile Warter

**Affiliations:** 1 Viral Diseases Branch, Walter Reed Army Institute of Research, Silver Spring, Maryland, United States of America; 2 Entomology Branch, Walter Reed Army Institute of Research, Silver Spring, Maryland, United States of America; 3 Department of Pathobiological Sciences, Louisiana State University, Baton Rouge, Louisiana, United States of America; 4 Veterinary Services Program, Walter Reed Army Institute of Research, Silver Spring, Maryland, United States of America; 5 GSK Vaccines, Rixensart, Belgium; 6 GSK Vaccines, Rockville, Maryland, United States of America; Centers for Disease Control and Prevention, UNITED STATES

## Abstract

Dengue virus (DENV) is transmitted by infectious mosquitoes during blood-feeding via saliva containing biologically-active proteins. Here, we examined the effect of varying DENV infection modality in rhesus macaques in order to improve the DENV nonhuman primate (NHP) challenge model. NHPs were exposed to DENV-1 via subcutaneous or intradermal inoculation of virus only, intradermal inoculation of virus and salivary gland extract, or infectious mosquito feeding. The infectious mosquito feeding group exhibited delayed onset of viremia, greater viral loads, and altered clinical and immune responses compared to other groups. After 15 months, NHPs in the subcutaneous and infectious mosquito feeding groups were re-exposed to either DENV-1 or DENV-2. Viral replication and neutralizing antibody following homologous challenge were suggestive of sterilizing immunity, whereas heterologous challenge resulted in productive, yet reduced, DENV-2 replication and boosted neutralizing antibody. These results show that a more transmission-relevant exposure modality resulted in viral replication closer to that observed in humans.

## Author summary

Dengue virus is transmitted into the skin of humans by mosquitoes as they take a blood meal. In contrast, many animal models are infected in the laboratory using a syringe to inject below the skin. Here, we looked at how different routes and methods of infection altered dengue infection in rhesus macaques. We found that infection via mosquito feeding resulted in a number of changes compared to other routes and methods, including a delay in the time to detection of dengue virus and overall greater quantities of dengue virus in the blood, and changes in the amounts of various components of blood that have been associated with dengue disease in humans. After 15 months, we exposed the macaques again to either the same or a different type of dengue virus. We found that animals exposed to the same type of dengue virus were protected from infection, whereas those animals exposed to a different type were only partially protected. Overall, our results show that dengue virus delivery using the natural transmission vector, mosquitoes, results in infection that is closer to what is observed in humans and may influence the interpretation of future studies of candidate vaccines.

## Introduction

Dengue virus (DENV) is a positive-sense RNA virus transmitted by mosquito vectors (primarily *Aedes* spp.) consisting of four, often co-circulating, serotypes in the genus flavivirus. Each serotype is comprised of multiple distinct genotypes, and include sylvatic genotypes that infect wild non-human primates (NHP). In humans, dengue is an acute febrile disease, i.e., classical dengue fever (DF), accompanied by rash, headache, eye pain, and arthralgia, with laboratory findings of transient neutropenia, AST and ALT elevations and, less frequently, thrombocytopenia.[1–5] During the febrile period circulating virus (viremia) or viral RNA (RNAemia) can often be detected by cell-based or genetic amplification assays, respectively. Approximately 3.2 million cases of DF were reported to the WHO in 2015, and unreported case estimates increase the annual global incidence to more than 50 million, including over 25,000 deaths due to the more severe forms of disease, dengue hemorrhagic fever (DHF) and dengue shock syndrome (DSS).[6] A major risk factor for DHF/DSS is the presence of serotype cross-reactive antibody after an initial or primary DENV infection, which in some individuals leads to enhanced viral replication after a second infection with a heterologous serotype.[7] This potential for immune-mediated enhancement of viral replication has complicated vaccine development efforts.

Aside from a few immune deficient murine models, macaques are the most widely accepted animal model for preclinical evaluation of dengue vaccine candidates, with DENV challenge typically administered by subcutaneous (SC) injection. For such models the endpoint is usually inapparent infection manifested by viremia, rather than overt disease.[8–18] As an example, the tetravalent dengue vaccine that has undergone the most comprehensive animal and clinical trials, Dengvaxia, exhibited >90% overall efficacy in vaccinated rhesus macaques challenged with each DENV serotype by the SC route, as measured by protection from viremia.[18] However, this vaccine was found to be approximately 60% efficacious overall in humans and, more concerning, dengue-seronegative children who received the vaccine were at greater risk for hospitalization and severe virologically confirmed dengue from subsequent DENV natural infection compared to unvaccinated children.[19–21] It is unclear why the macaque model in this instance failed to demonstrate this limitation of the vaccine. Critical differences observed between pre-clinical and field efficacy and safety data for Dengvaxia underscore the need for a more predictive animal model for testing future dengue vaccine candidates.

Previously, we hypothesized that the limited utility of the rhesus macaque for accurately predicting vaccine efficacy may relate to a number of factors such as the use of high-passage, non-contemporary DENV strains, the relatively low viremia levels observed in macaques, and assessment of vaccine efficacy based mainly on the degree of reduction in post-challenge DENV replication markers (e.g., viremia or RNAemia) while neglecting other biological parameters associated with human dengue infections, which may be considered as proxies for clinical disease. To address these limitations, we demonstrated that a low cell-passage level, contemporary DENV clinical isolate from Brazil induced viremia of higher magnitude and longer duration compared to previously reported viremia kinetics in macaques, and also changes in cytokine profiles similar to those in patients with acute DF.[22] Furthermore, we showed that when assessing vaccine efficacy by combining viremia and RNAemia measurement with characterization of changes in relevant biological markers, safety signals associated with a dengue vaccine could be detected in the SC-inoculated macaque model.[23] However, despite these improvements, the model did not demonstrate clinical signs, and viremia/RNAemia, onset of which occurred 1–2 days post-challenge, remained low both in terms of magnitude and duration compared to viremia/RNAemia reported in severe dengue patients.

Previous attempts to develop a dengue disease model in NHP have employed intravenous (IV) administration of very high challenge virus doses, atypical routes for challenge (e.g., intramuscular injection) which are far removed from the conditions of natural vector-mediated infection, and the utilization of non-macaque species which in some cases demonstrate disease signs such as transient rash, coagulopathy, or elevated liver enzymes. However, results from such studies are often difficult to reproduce, possibly due to unrecognized variables, and most did not include comparative SC inoculation controls.[24–26] In macaques, comparisons to SC challenge of alternative routes of administration have been evaluated. Macaques challenged with DENV-2 by the intradermal (ID) route seroconverted more rapidly than those challenged by the SC route [27], and macaques challenged with DENV-2 by the IV route exhibited higher peak RNAemia titers compared to those challenged ID or SC.[28] Small groups of macaques infected with DENV-3 by the SC, ID, or IV routes exhibited some differences in the tissue distribution of virus, but all groups exhibited only transient (1–4 days) and low level viremia.[29]

The effect of prior DENV exposure or immunity on subsequent DENV infection has also been explored in macaques. These studies utilized sequential infections and passive transfer of sub-neutralizing concentrations of pooled, human DENV-immune sera or DENV-specific monoclonal antibody to demonstrate significantly higher DENV viremia titers compared to non-immune controls.[30–32] Additionally, some animals and DENV serotypes in the sequential infection study exhibited reduced viremia, indicating some level of cross-protection.[30] Together, these studies indicate that, as observed in humans, heterologous DENV infection and waning or insufficient humoral immunity can be associated in NHP with increased DENV replication.

As the effect of the natural mosquito vector and/or mosquito salivary proteins (MSP) on the course of DENV infection has not been rigorously studied in NHP, it is possible that the mosquito vector plays an essential role in determining the outcome of infection in terms of primary target cell(s), virus dissemination pathways, and immune activation, all of which may impact disease severity. Blood-feeding by mosquitoes on vertebrate hosts is initiated by probing, which results in physical damage to the epithelium and vasculature as well as simultaneous introduction of virus and MSP into the cutaneous layers of the skin.[33–36] MSP contains biologically active proteins that modulate host hemostasis and immune responses, which in turn facilitate blood-feeding and virus transmission.[37–39] The effects of MSP on mosquito-borne viral infections have been reviewed previously, and include 1) alterations to vascular permeability in DENV infection, 2) changes in cytokine and innate signaling profiles, such as decreased expression of IFNβ/γ, IL-2, and TLR3/7 and increased expression of IL-4, IL-10, and IL-12 in DENV, Chikungunya virus (CHIKV), and West Nile virus (WNV) infections, and 3) migration of monocytes, neutrophils, eosinophils, and plasma cells to the site of infection for DENV, CHIKV, and WNV, as well as increased WNV neurovirulence.[38, 40–47] Additionally, *Aedes aegypti* MSP increased the prevalence of disease signs and extended the viremic period in DENV-infected, humanized mice.[48] Various methods have been utilized to incorporate MSP into arbovirus research, including salivary gland extract (SGE) and feeding by infectious mosquitoes.[40–42, 47–58] While the use of feeding mosquitoes offers the most naturally relevant exposure method, SGE allows for greater control over, and reproducibility of, the administered dose.[59]

In an attempt to further increase the clinical relevance of the dengue macaque model, we compared different viral infection modalities including live mosquito vector-mediated, ID inoculation of virus with and without MSP, and conventional SC viral inoculation, all using low-passage, i.e., near wild-type, contemporaneous DENV clinical isolates. In order to reduce the impact of variation in past exposure to MSP antigens, all animals in the study were primed by multiple, sequential exposures to uninfected, feeding *Ae*. *aegypti* before being exposed to DENV. We then examined viral replication magnitude/kinetics and host immune responses following primary infection with DENV-1, and thereafter, following secondary infection with either DENV-1 (homologous) or DENV-2 (heterologous).

## Results and discussion

Dengue virus (DENV) is naturally transmitted by virus-infected mosquitoes in regions where *Ae*. *aegypti* are endemic and post-primary DENV infections are common. Accordingly, animal models that are utilized in investigations of DENV pathogenesis and the evaluation of dengue vaccine candidates should take these important factors into consideration. We hypothesized that infection modalities closer to natural, mosquito vector-mediated viral transmission may result in viral replication kinetics and elicit other infection and disease markers more like those seen in humans. In this experiment, all NHP were exposed multiple times to MSP via blood-feeding by naïve, laboratory-reared *Ae*. *aegypti* in order to provide exposure of all NHP to a common population of mosquitoes. The long-standing Rockefeller colony of *Ae*. *aegypti* was chosen for this study instead of a recent generation of field-caught mosquitoes because, as a more homogenous population of indiscriminate feeders, it is more likely to promote reproducibility of the model. Groups of these NHP were later infected with DENV-1 by one of four methods (Table 1): standard SC injection, ID injection with and without mosquito salivary gland extract (SGE), and infectious mosquito feeding (IMF). After an interval of 15 months, we exposed subsets of these macaques to homologous or heterologous serotypes of DENV in order to assess the model in the context of post-primary infections.

**Table 1:** Experimental groups and study design.

Primary infection				15 month interval	Secondary infection			
Group / Infection Method	N	Virus†	Inoculum per animal^#^		Group / Infection Method	N	Virus†	Inoculum per animal^#^
SC	10	DENV-1	5.18 log_10_ PFU		SC D1-D1	5	DENV-1	5.18 log_10_ PFU
					SC D1-D2	5	DENV-2	5.18 log_10_ PFU
ID	9	DENV-1	5.18 log_10_ PFU		SC N-D2	5	DENV-2*	5.18 log_10_ PFU
ID+SGE	10	DENV-1	5.18 log_10_ PFU + 15 SGeq		IMF N-D2	5	DENV-2*	15 mosquitoes
IMF	10	DENV-1	15 mosquitoes		IMF D1-D1	5	DENV-1	15 mosquitoes
					IMF D1-D2	5	DENV-2	15 mosquitoes

*primary DENV2 infection controls for the SC injection and IMF methods

†DENV-1 strain 0111/2011; DENV-2 strain 0126/2010

^#^IMF animals were exposed to the probing and/or feeding of 15 mosquitoes each; details of the inoculum are available in the “Materials and Methods” and in S4 Table.

SC = subcutaneous; ID = intradermal; SGE = salivary gland extract; IMF = infectious mosquito feeding; SGeq = salivary gland equivalents in PBS

### Primary DENV-1 inoculation of rhesus macaques via IMF resulted in delayed and increased viral replication kinetics compared to other infection modalities

**Fig 1:**
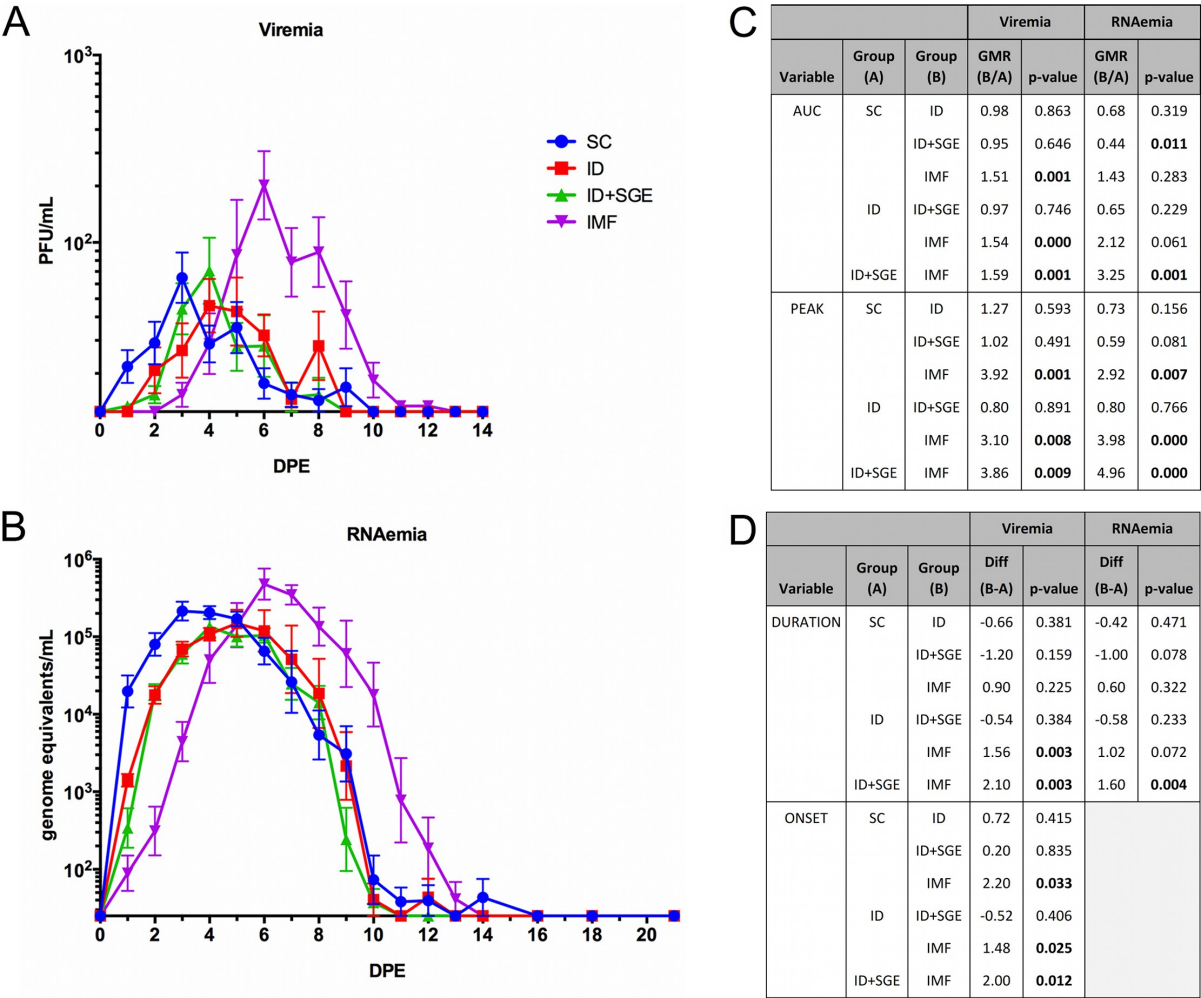
Differential viremia and RNAemia profiles after primary DENV-1 infection via subcutaneous, intradermal (with or without salivary gland extract) or infectious mosquito feeding exposure. Sera were collected daily following DENV-1 exposure and tested, in duplicate, by A) plaque assay for their infectious virus (viremia) titer expressed as plaque forming units (PFU)/mL, and by B) qRT-PCR for their RNAemia titer expressed as genome equivalents (GE)/mL. Geometric mean titers with standard error of the log_10_-transformed mean are displayed. C) Geometric mean ratios (GMR) and p-values for between-group comparisons of viremia and RNAemia areas under the curve (AUC) and peak titers are shown. AUC and peak titers were log_10_-transformed for statistical analysis using an ANOVA model. Peak titers were additionally compared using a non-parametric analysis (ANOVA on ranks) to confirm the log_10_-transformed results. D) Viremia and RNAemia times to onset and durations were compared between groups using an ANOVA model, with Least Squares means differences and p-values shown. n = 10 for each SC, ID+SGE, and IMF. n = 9 for ID. *p*<0.05 are in bold text. SC, subcutaneous; ID, intradermal; ID+SGE, intradermal + salivary gland extract; IMF, infectious mosquito feeding; DPE, day post exposure.

Macaques infected by mosquito (IMF group) demonstrated peak serum infectious virus (viremia) and viral RNA (RNAemia) titers that were significantly higher than those achieved by SC, ID, and ID+SGE infection modalities (3.1–3.92- and 2.92–4.96-fold, respectively; Fig 1). The duration of viremia for the IMF group was also significantly longer compared to the ID and ID+SGE groups, but not the SC group; whereas, the duration of RNAemia for the IMF group was significantly longer than for the ID+SGE group, but not the SC and ID groups. Considering both the magnitude and the duration of viremia estimated by the area under the curve (AUC), AUC was also significantly greater for the IMF group compared to the other three groups (>1.5-fold). And, although RNAemia AUCs were similar between the IMF and SC groups, these were significantly greater than that of the ID+SGE group; the RNAemia AUC for the ID group was not significantly different from other groups. Mean time to detection (onset) of viremia for the IMF group was 4.7 days, which was significantly longer compared to the other three groups by 1.5–2.2 days.

Together, these results demonstrated that DENV-1 replication kinetics were delayed and increased by IMF, in terms of both AUC and peak magnitude, when compared to the other modalities for virus delivery. As such, the timing of DENV presence and peak in the peripheral blood of the IMF group macaques more closely resembles the published dengue human infection model (average viremia onset day 4.7) than do the SC group macaques (average viremia onset day 2.5, in agreement with previous estimates).[60–63] Surprisingly, the addition of SGE to the ID injection route did not result in the same kinetics observed in the IMF group, suggesting that direct mosquito delivery differs from simple inclusion of salivary gland components in a DENV inoculum.

Importantly, we cannot discount potential differences in delivered doses between the IMF and needle-inoculated groups as a contributing factor to these results. The estimated dose of DENV delivered by a mosquito has not been extensively investigated, and it may also depend on the strains of mosquito and virus used and the method by which the quantity is determined. One estimate of DENV-2 expectorated by *Ae*. *aegypti* into capillary tubes determined a maximum of approximately 10 PFU or 10^4^ genome equivalents.[48] Another estimate of a different strain of DENV-2 expectorated by *Ae*. *albopictus* into a suspended droplet measured a range of approximately 10^2.5^–10^5.2^ mosquito infectious doses_50_, which would translate to a minimum of 10^2.2^–10^4.9^ infectious virions.[64] This latter estimate is more consistent with the needle-inoculated dose used here (10^5.18^ PFU) and with estimates of West Nile virus transmission during feeding on animals, which ranges from 10^3.6^–10^6.1^ PFU depending on the mosquito species tested.[34]

### Higher elevations of liver aminotransferase were observed in IMF and ID+SGE compared to SC and ID groups

**Fig 2:**
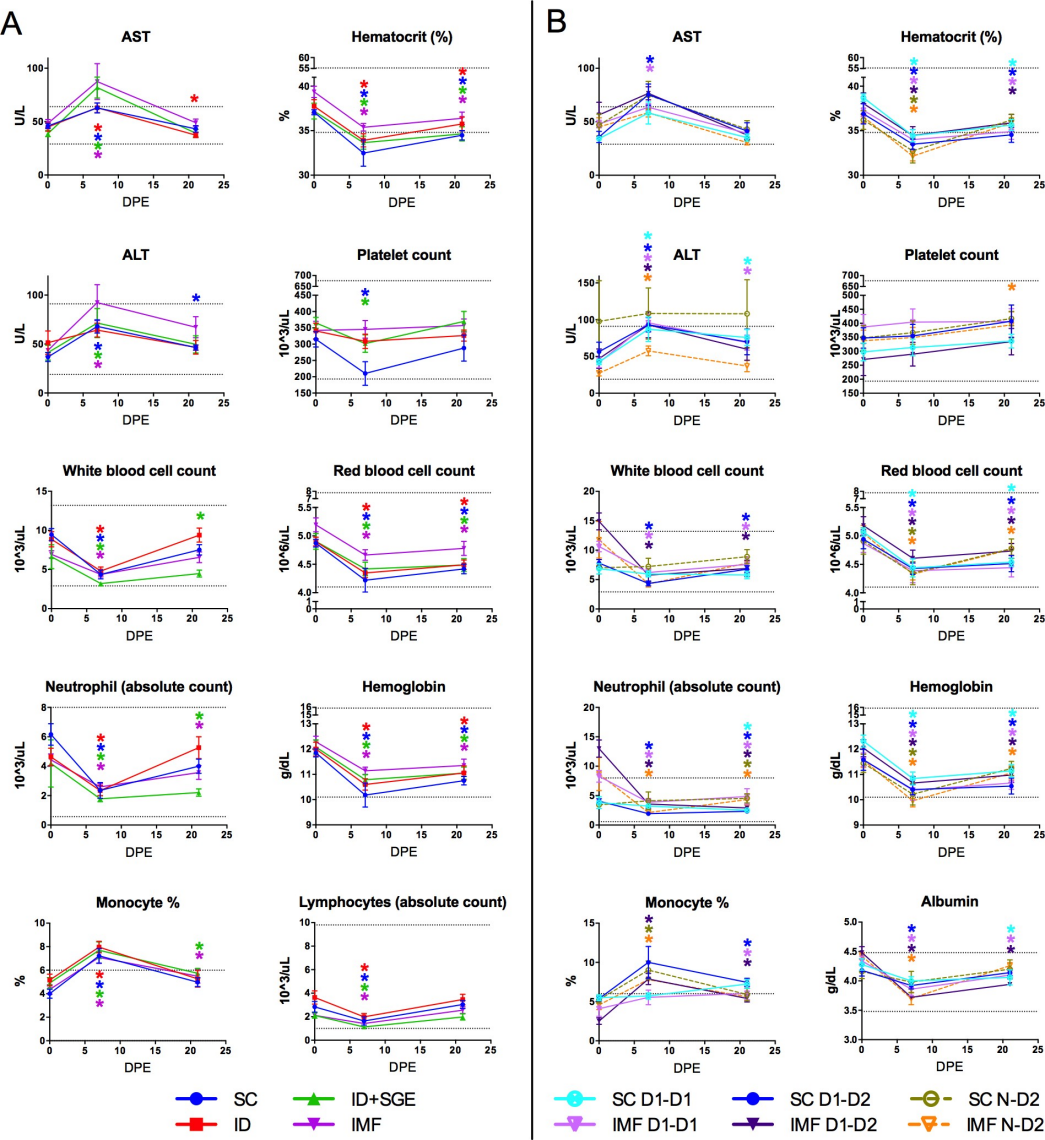
Hematology and clinical chemistries following primary and secondary DENV exposures. Data were measured on study days 0, 7, and 21 post A) primary DENV-1 infection via different routes/modalities and B) homologous DENV-1 re-exposure, heterologous DENV-2 infection, and primary DENV-2 infection. Analyses were based on an ANOVA model on the change from baseline with the covariates baseline value of the variable and baseline weight and group as factors. n = 10 for each SC, ID+SGE, and IMF; n = 9 for ID; n = 5 for the remainder of groups. (*, *p*<0.05). SC, subcutaneous; ID, intradermal; ID+SGE, intradermal + salivary gland extract; IMF, infectious mosquito feeding; D1-D1, homologous DENV-1; D1-D2, heterologous DENV-2; N-D2, primary DENV-2.

Thrombocytopenia, leukopenia, decreases in lymphocyte, monocyte, and neutrophil counts, elevated percent monocytes, and elevated levels of aminotransferases are considered biological hallmarks of dengue fever (DF) in humans.[2–5, 65] To determine if these hematologic and serum chemistry parameters were impacted by infection modality, we evaluated blood and serum samples collected before, during, and after primary DENV-1 infection (days 0, 7, 21; Figs 2A and S1A). Similar to DF patients, all groups demonstrated a significant decrease in absolute white blood cell (WBC), lymphocyte, and neutrophil counts, whereas differentials showed an increase in percent monocytes, on day 7. There was also an increase in AST on day 7 compared to baseline levels for all groups, and in ALT for all except the ID group. Significant decreases in RBC count, hemoglobin, and hematocrit (HCT) relative to baseline values were observed in all groups on day 7, as well as a significant increase in reticulocyte counts for all groups on day 21, the latter of which is likely attributable to recovery from daily blood collections.

Between-group comparisons revealed that absolute WBC counts on day 7 were significantly lower for the ID+SGE group versus the ID and IMF groups. Neutrophil counts were also significantly lower for this group compared to the IMF group. Mean AST levels above the upper limit of normal were observed for the ID+SGE and IMF groups, but only for the ID+SGE group was the mean AST level significantly higher than that observed for the SC and ID groups. Although the mean platelet counts fell below baseline for the SC and ID+SGE groups they remained within the normal reference range. Nevertheless, the mean platelet count for the SC group was still significantly lower than that for the IMF group. Additional significant changes in mean values from baseline and significant differences between groups are presented in S2 Table. Importantly, we cannot exclude that differences might have been detected at other time points post-challenge, particularly given the differences in DENV replication kinetics described above.

### Skin inflammation/immune cell infiltration was detected at the site of DENV-1 administration in ID+SGE and IMF groups

Administration site skin biopsies were collected prior to primary DENV-1 exposure as well as at 6h, 24h and 21 days post-exposure, and examined for local inflammation (i.e. dermatitis/panniculitis) and immune cellular infiltration. Each biopsy sample was scored from 0–4, with 0 corresponding to a lack of local inflammation/cellular infiltration, and 4 corresponding to marked inflammation/cellular infiltration (S2A Fig). None of the animals from the SC group scored above baseline after DENV-1 exposure, and only 1/9 animals from the ID group scored above baseline, with a score of 2 at 6h post-exposure. In contrast, in the ID+SGE group, 9/10 animals had a score of 1 or 2 at 6h post-exposure and 8/10 animals had a score of 1 or 2 at 24h post-exposure. Similarly, in the IMF group, 3/10 animals had a score of 1 or 2 at 6h post-exposure and 7/10 animals had a score of 1 or 2 at 24h post-exposure. Cumulatively, 19/20 animals from ID+SGE and IMF groups showed skin inflammation/cellular infiltration within 24h of DENV-1 exposure, albeit at minimal to mild levels, compared to only 1/19 animals from the virus-only groups. Altogether, these data implicate mosquito-based factor(s) as the impetus for this inflammation.

### IMF induced expression of Th2 cytokines and not of double-stranded RNA pattern recognition receptors at the site of DENV-1 administration

**Fig 3:**
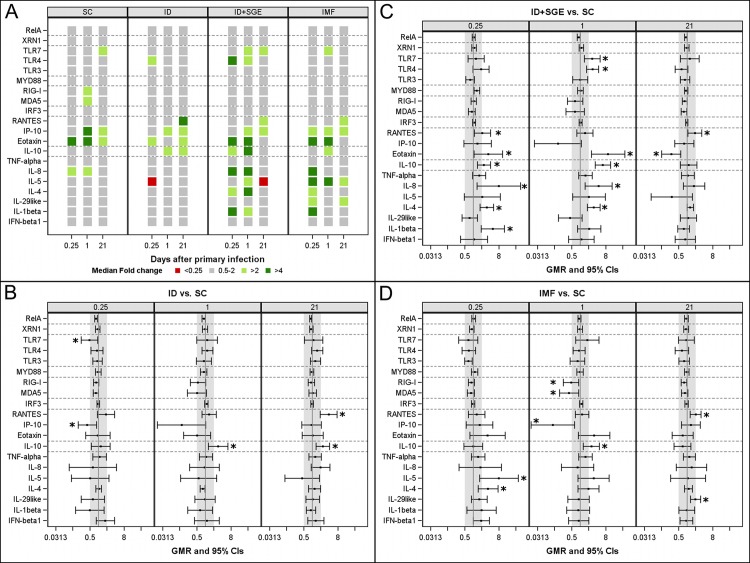
Transcriptional analysis following primary DENV-1 infection in administration sites of skin. Skin biopsies from DENV-1 administration sites were collected immediately prior to administration (0h), and at 6 hours, 24 hours, and 21 days later. A) Heat map of median fold changes from baseline (0h) by group and time point for each gene. B), C), and D) display geometric mean ratios with 95% confidence intervals of ID, ID+SGE, and IMF groups, respectively, relative to the SC group. Vertical grey bands indicate the 2-fold cutoff region on either side of 1 (no change). Analyses of transcriptional data were based on fold change from baseline derived from the ΔΔCt method. To compare each group to the SC group, an ANOVA model was fitted on log_2_ fold changes by including group, time, and group*time as fixed effects. Time was considered as a repeated factor with an unstructured matrix and heterogeneous variances between groups were assumed. Geometric means and geometric mean ratios and their respective 95% CIs were derived from this model. No adjustment for multiplicity was performed. n = 10 for each SC, ID+SGE, and IMF; n = 9 for ID. (*, *p*<0.05). SC, subcutaneous; ID, intradermal; ID+SGE, intradermal + salivary gland extract; IMF, infectious mosquito feeding.

To assess whether DENV-1 infection modality impacted early immune responses at the site of administration, transcriptional analyses were conducted on skin biopsy samples from these sites. RNA transcript median expression levels are shown by group and time point in Fig 3. In all groups, expression was elevated over baseline for eotaxin, which is chemotactic for eosinophils, at 6h and for IP10, which is chemotactic for mononuclear cells, at 24h and 21d. Expression of IL-10, a pleotropic immunomodulatory cytokine best known for inhibiting IFNγ and type 1 inflammation, was elevated in all groups except for SC at 24h. Expression levels of RIG-I and MDA5, two double-stranded cytoplasmic RNA pattern recognition receptors (PRRs), were elevated only in the SC group at 24h. Expression levels of IL-1β, IL-4, and IL-5 were elevated only in the two mosquito-related groups (ID+SGE and IMF).

Statistical difference was assessed for each group relative to the SC group. In the ID group, TLR7 and IP10 expression was significantly lower than in the SC group at 6h, and IL-10 expression was significantly higher at 24h and on day 21. In the ID+SGE group relative to the SC group, expression levels of IL-4, IL-8, IL-10, and eotaxin were significantly elevated at 6h and 24h; IL-1β and RANTES at 6h and TLR4 and TLR7 at 24h were also significantly elevated. IMF induced significantly increased transcription of the traditionally T_H_2-associated cytokines IL-4 and IL-5 at 6h, followed by elevated IL-10, decreased IP10, and decreased RIG-I and MDA5 at 24h compared to the SC group. Taken together, the transcriptional responses observed here would indicate an IMF-induced shift away from the early, anti-viral response observed with SC inoculation of DENV-1 at the site of initial replication, similar to previous observations from murine models.[40, 44, 66, 67]

### Serum cytokines vary by DENV-1 administration route

**Fig 4:**
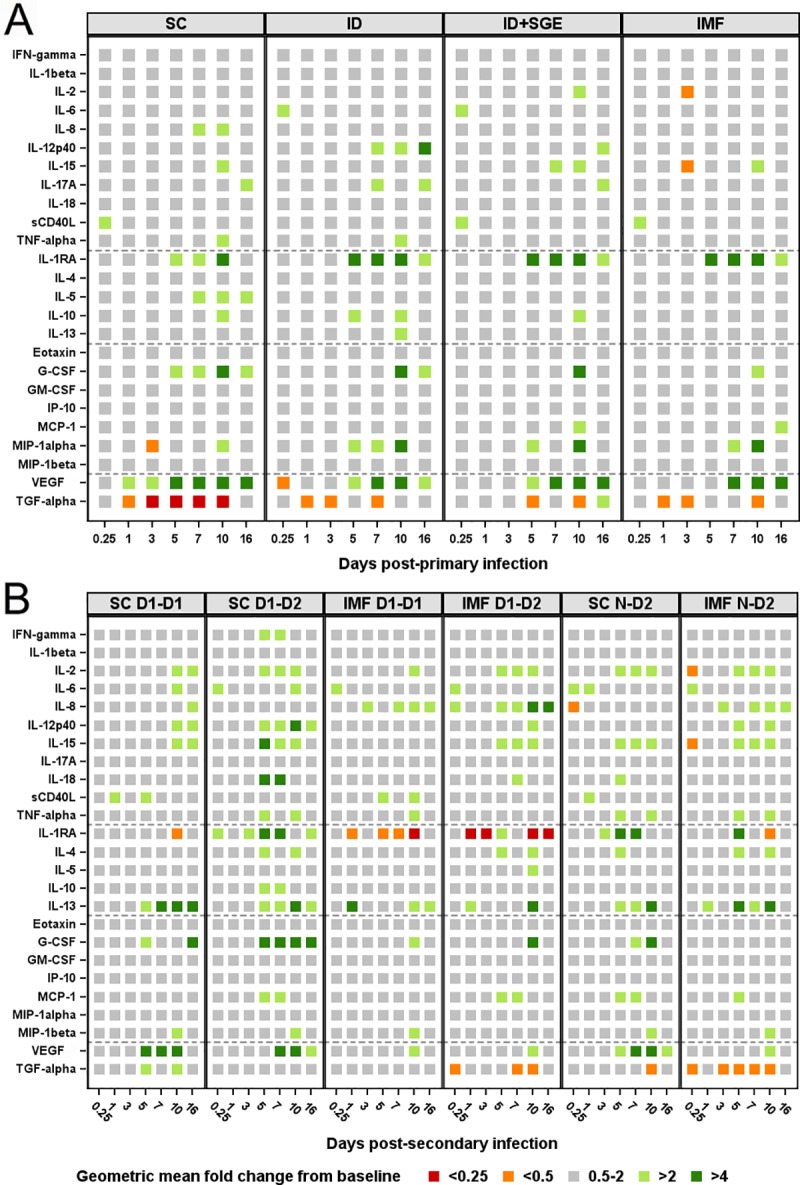
Cytokine protein detection in peripheral blood following primary and secondary DENV exposures. Serum was collected at 0 hour, 6 hours, and days 1, 3, 5, 7, 10, and 16 post A) primary DENV-1 infection and B) homologous DENV-1 re-exposure, heterologous DENV-2 infection, and primary DENV-2 infection. Twenty-five cytokines, chemokines, and growth factors in serum were assessed. Data are expressed as geometric mean fold change ranges relative to baseline (0h). Fold change values within 2-fold of baseline were considered unchanged from baseline. n = 10 for each SC, ID+SGE, and IMF; n = 9 for ID; n = 5 for the remainder of groups. SC, subcutaneous; ID, intradermal; ID+SGE, intradermal + salivary gland extract; IMF, infectious mosquito feeding; D1-D1, homologous DENV-1; D1-D2, heterologous DENV-2; N-D2, primary DENV-2.

To assess whether a parallel exists between the immune transcript expression detected in the administration site skin and systemic cytokine production evident in peripheral blood, 25 cytokines, chemokines, and growth factors in serum were assessed following primary infection with DENV-1 (Figs 4A and S3). Cytokine production profiles in serum were not observed to be comparable to the transcript expression observed in the administration site biopsies for the parameters present in both assays. However, we did observe cytokine production outside of the 2-fold cutoff range specific to certain groups. For example, IL-5 and IL-8 were elevated in only the SC group, whereas IL-6, IL-17, and IL-12/23p40 were elevated only in the ID and ID+SGE groups, and MCP1 was elevated in only the ID+SGE and IMF groups. None of the cytokines tested were expressed outside the 2-fold range exclusively in the IMF group. In contrast, IL-2 and IL-15 were reduced exclusively in the IMF group, which preceded the day of peak RNAemia. The synchronous decrease in both IL-2 and IL-15 is notable as both cytokines utilize the same heterodimeric cytokine receptor (IL-2Rβ/IL-2Rγ), and are critical proliferative and pro-survival factors for activated T cells. Interestingly, the onset of elevated VEGF, a cytokine involved in angiogenesis and vascular permeability, coincides with the day of peak RNAemia at the group level in all groups; elevated VEGF on early days of illness has been proposed as a predictor of severe dengue in humans.[68] Due to the disparity between administration site responses and serum cytokine production, and the systemic focus of the remainder of the assessments herein, administration site transcriptional analyses were not conducted in the latter phase of the study.

### Cross-reactive neutralizing antibody was detected consistently without detectable waning following primary DENV-1 infection

**Fig 5:**
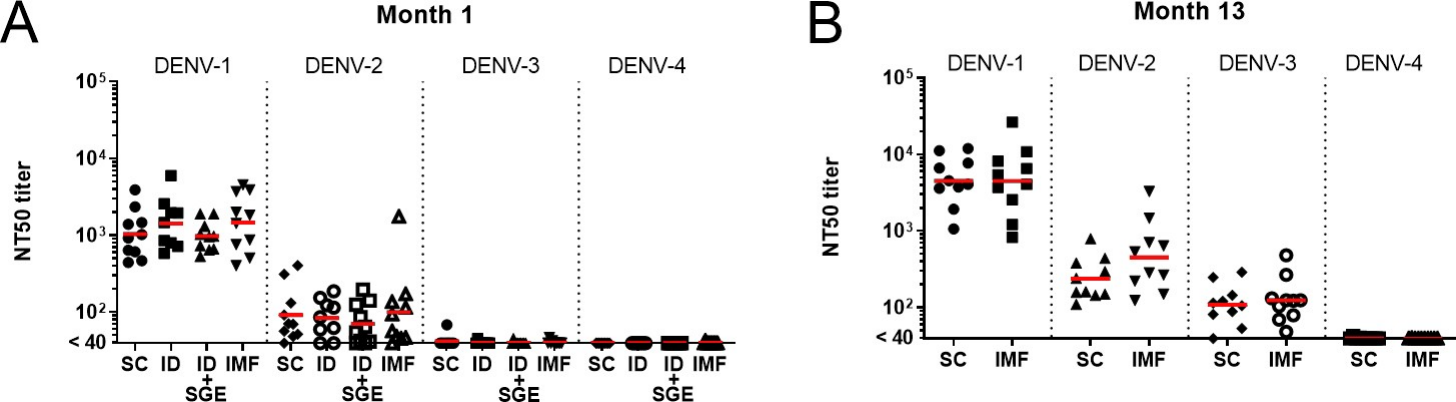
Neutralizing antibody titers following primary DENV-1 infection. Geometric mean NT50 titers (red bars) and individual data points for A) month 1 and B) month 13 post infection sera are shown for each of the four DENV serotypes. To compare IMF to the SC group, an ANOVA model was fitted on log_10_ fold changes by including group, time, and group*time as fixed effects. Time was considered as a repeated factor with an unstructured matrix and heterogeneous variances between groups were assumed. Geometric means and geometric mean ratios and their respective 95% CIs were derived from this model. No adjustment for multiplicity was performed. No differences were found between groups at these time points for NT50 titer. n = 10 for each SC, ID+SGE, and IMF; n = 9 for ID. SC, subcutaneous; ID, intradermal; ID+SGE, intradermal + salivary gland extract; IMF, infectious mosquito feeding.

Neutralizing antibody titers against the four DENV serotypes were assessed, in both the SC and IMF groups, at months 1, 5, 8, 10, and 13 post-primary DENV-1 exposure. Geometric mean NT50 titers for months 1 (all groups) and 13 (SC and IMF groups only) are shown in Fig 5 and longitudinal NT50 titers across all time points are shown for individual animals from the SC and IMF groups in S4 Fig. Month 1 NT50 titers were highest against the infecting DENV-1 serotype, moderate against DENV-2, and low to undetectable against DENV-3 and 4, irrespective of group. Neutralizing antibody titers continued to develop over time, with a moderate 3-4-fold rise in geometric mean titers against DENV-1 and DENV-2 between month 1 and month 13. Additionally, most NT50 titers against DENV-3 were undetectable (<40) at month 1 and rose to geometric mean titers of over 100 at month 13. A similar lack of waning, cross-neutralizing anti-DENV antibody in rhesus macaques has been reported previously.[26]

The ability of serially diluted month 13 sera to promote antibody-dependent infection enhancement (ADE) in vitro of the DENV-2 strain 0126/2010 used for secondary infections was also assessed. There was approximately a 15-fold increase in mean peak infection in the presence of this DENV-1-primed sera relative to negative control sera from both the SC and IMF groups. However, no statistical difference was observed between the two groups. ADE curves with NT50 overlaid for each animal are shown in S5 Fig. All animals had a detectable NT50 titer to DENV-2 and the ADE peak was observed at dilutions at or greater than the NT50 indicating that undiluted serum in these animals would be expected to neutralize virus infectivity and not mediate ADE.

### Primary DENV-2 replication kinetics differ from those of primary DENV-1

**Fig 6:**
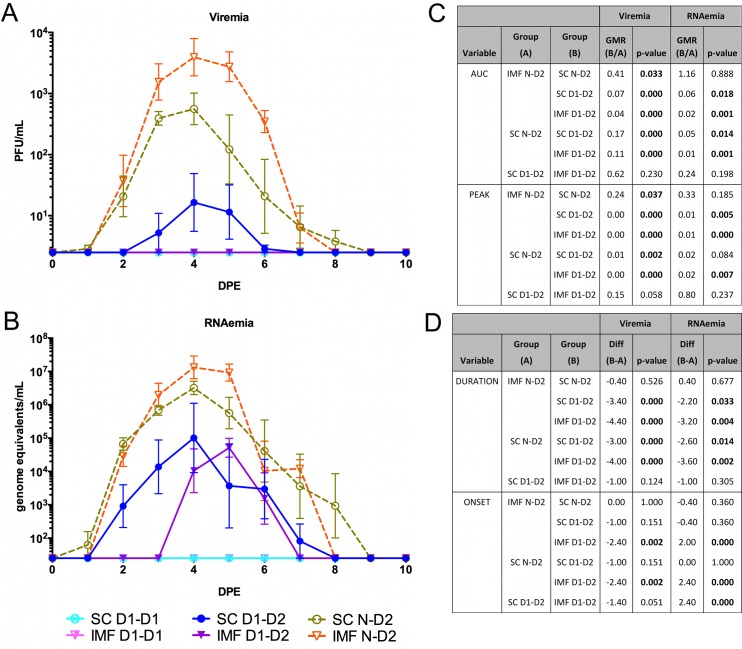
Comparative viremia and RNAemia profiles associated with homologous versus heterologous secondary DENV infection. At month 15 post primary DENV-1 infection, the SC and IMF groups were divided into two subgroups, each of which were exposed to either DENV-1 or DENV-2 using the same administration route that was used for their respective primary infections. In parallel, DENV-naïve macaques were exposed to DENV-2, either via SC inoculation or IMF, as controls for primary DENV-2 infection. Sera were collected daily following DENV exposure and tested, in duplicate, by A) plaque assay for their infectious virus (viremia) titer expressed as PFU/mL, and by B) qRT-PCR for their RNAemia titer expressed as genome equivalents/mL. Geometric mean titers with standard error of the log_10_-transformed mean are displayed (n = 5/subgroup). C) Geometric mean ratios (GMR) and p-values for between group comparisons of viremia and RNAemia areas under the curve (AUC) and peak titers are shown. AUC and peak titers were log_10_-transformed for statistical analysis using an ANOVA model. Peak titers were additionally compared using a non-parametric analysis (ANOVA on ranks) to confirm the log_10_-transformed results. D) Viremia and RNAemia times to onset and durations were compared between groups using an ANOVA model, with LS means differences and p-values shown. SC, subcutaneous; IMF, infectious mosquito feeding; D1-D1, homologous DENV-1; D1-D2, heterologous DENV-2; N-D2, primary DENV-2.

As shown in Table 1, 15 months after primary infection, the SC and IMF groups were split into two subgroups of five animals each and homologously re-exposed to the same strain of DENV-1 (SC D1-D1 and IMF D1-D1) or heterologously exposed to DENV-2 (SC D1-D2 and IMF D1-D2). Two additional groups of 5 animals each were included as infection modality controls for DENV-2 exposure in flavivirus-naïve macaques (SC N-D2 and IMF N-D2). As in the primary DENV-1 infection groups, serum RNAemia and viremia were measured and statistical comparisons were made for peak titer observed, AUC, day of onset, and duration. Interestingly, as shown in Fig 6, the IMF N-D2 group did not differ significantly in RNAemia peak, AUC, onset, or duration as compared to SC N-D2, yet did demonstrate a significantly higher viremia peak titer (4.17-fold) and AUC (2.44-fold). The discrepancies in the IMF and SC observations between primary DENV-1 and DENV-2 infections suggest a serotype- or strain-specific relationship with the infection modality, possibly through differences in vertebrate or vector infectivity or through differences in interactions with specific MSP. Serotype differences in the onset and duration of viremia in NHP have been identified previously, suggesting that this IMF model is likely also useful for investigating these differences.[69] In addition, specific interactions between individual MSP and DENV have been described previously, and utilizing a model that allows for these interactions may prove critical in studies of DENV pathogenesis and vaccine development.[69]

### Secondary exposure resulted in partially or completely controlled DENV replication

Heterologous exposure to DENV-2 differed from primary infection with DENV-2 (Fig 6). In opposition to our study hypothesis that viremia/RNAemia would be unchanged or increased during heterologous infection compared to primary infection, many viremia/RNAemia parameters were reduced or not detected in secondary DENV-2 SC (SC D1-D2) and secondary DENV-2 IMF (IMF D1-D2) compared to the corresponding primary DENV-2 SC and IMF groups. The duration of RNAemia for SC D1-D2 and IMF D1-D2 groups was significantly shortened by 2.6 and 3.2 days, respectively, and the RNAemia AUC was significantly less in these groups, as compared to primary DENV-2 infections. Also, there was a significant 162-fold decrease in peak RNAemia of the IMF D1-D2 group relative to primary DENV-2 IMF. These data may be explained by the unexpectedly long-lasting and high titer DENV-2 cross neutralizing activity resulting from primary DENV-1 infection.

As in primary DENV-2 infection, RNAemia peak, AUC, and duration did not differ significantly between IMF D1-D2 and SC D1-D2. In contrast, RNAemia onset in IMF D1-D2 was significantly delayed relative to SC D1-D2. Unlike primary DENV-2 IMF, IMF D1-D2 did not demonstrate detectable viremia as measured by plaque titration. Importantly, on an animal by animal basis, we consistently observed plaque production only in serum samples with genome equivalents/mL of at least 545,010 in secondary DENV-2 infections; none of the animals in IMF D1-D2 had genome equivalents/mL peaks that reached this threshold. Not surprisingly, no replication of DENV-1 in homologous re-exposure groups was observed in the periphery.

### Both SC and IMF secondary exposures exhibited hematological and biochemical parameter shifts consistent with human DENV infection

As with primary DENV-1 exposures, hematology and clinical chemistries were assessed on study days 0, 7, and 21 post primary DENV-2, homologous DENV-1, and heterologous DENV-2 exposures (Figs 2B and S1B). Similar to primary DENV-1 exposures, HCT decreased and percent monocytes increased significantly in both SC N-D2 and IMF N-D2 groups on day 7 relative to baseline. Contrary to primary DENV-1 exposures, WBC counts in SC N-D2 and IMF N-D2 did not change significantly from baseline. Additionally, day 7 lymphocyte and neutrophil counts decreased and the levels of ALT increased significantly relative to baseline in IMF N-D2 but not in SC N-D2, whereas these changes extended to both SC and IMF groups after primary DENV-1 exposures. With regard to homologous secondary exposures on day 7, WBC and neutrophil counts decreased and AST levels increased significantly relative to baseline in IMF D1-D1 but not in SC D1-D1. With regard to heterologous secondary exposures on day 7, WBC counts and neutrophil counts decreased significantly relative to baseline in both SC D1-D2 and IMF D1-D2, lymphocyte counts decreased and AST levels increased significantly in SC D1-D2, and percent monocytes increased significantly in IMF D1-D2. As seen after primary DENV-1 exposure, significant decreases in RBC counts, hemoglobin, and HCT relative to baseline were detected in all groups on day 7.

Between-group comparisons revealed more significant differences reflective of administration route rather than exposure history. WBC counts and monocyte counts were significantly lower and percent lymphocytes higher in IMF N-D2 relative to SC N-D2. Albumin was significantly lower in IMF N-D2 compared to SC N-D2, as well as IMF D1-D2 relative to SC D1-D2; a reduction of albumin concentration has been suggested as a predictor of DHF in humans.[70] Lymphocyte counts were significantly lower in SC D1-D2 relative to IMF D1-D2. Comparing heterologous versus homologous secondary exposures, percent monocytes and basophils were significantly higher in SC D1-D2 relative to SC D1-D1. Lastly, percent basophils was significantly higher in SC D1-D2 relative to SC N-D2. All other significant mean changes from baseline and significant differences between groups are presented in S2 Table.

### Mild skin inflammation without clear association with a specific DENV infection sequence or modality

Administration site skin biopsies were again collected prior to DENV exposure (0h), and at 6h, 24h, and 21 days post-exposure, and examined for local inflammation (i.e. dermatitis/panniculitis) and immune cellular infiltration as above. Analysis revealed dermatitis/panniculitis scores above baseline in only 14 of 120 skin biopsy specimens (S2B Fig). None of the animals from SC D1-D1 scored above baseline, whereas 3/5 animals in IMF D1-D1 had a score of 1 or 2 at 6h and 1/5 had a score of 1 at 24h after DENV-1 re-exposure. Three of 5 animals in SC D1-D2 had a score of 1 or 2 at 24h, and 2/5 animals in SC N-D2 had a score of 1 or 2 at 24h. Similarly, 2/5 animals in IMF D1-D2 had a score of 1 or 2 at 24h and 1/5 had a score of 2 at 21d, and 2/5 animals in IMF N-D2 had a score of 1 or 2 at 24h.

### Serum cytokines vary by DENV serotype and infection modality

Serum cytokines were assessed following primary DENV-2, homologous DENV-1, and heterologous DENV-2 exposures as performed after primary DENV-1 infection (Figs 4B and S3). IL-4 and MCP-1 were elevated relative to baseline in the four groups receiving DENV-2 exposures but not in the homologous DENV-1 re-exposure groups; elevated production of these cytokines began on or immediately following the day of peak viremia/RNAemia. IFN-γ was elevated only in SC D1-D2, also beginning after peak RNAemia/viremia. IL-8 was elevated only in the three groups exposed by IMF. As observed in the primary DENV-1 IMF group, IL-2 and IL-15 exhibited decreased concentration in only the IMF N-D2 group, though at the earlier time point of 6h. VEGF exhibited elevated production at multiple time points only in the three SC exposure groups; in contrast to primary DENV-1 infection, the timing of VEGF production did not associate with the day of peak RNAemia in any discernable pattern.

### Neutralizing antibody titers following homologous DENV-1 re-exposure were indicative of sterilizing immunity

Neutralizing antibody titers against the administered serotypes were assessed on day 35 following secondary DENV and primary DENV-2 exposure. Geometric mean NT50 titers (GMT) were 426 and 636 in the SC N-D2 and IMF N-D2 groups, respectively, following primary DENV-2 exposure (S3 Table). GMT for animals in the SC D1-D2 and IMF D1-D2 groups increased 13.96- and 26.33-fold, respectively, following secondary, heterologous DENV-2 exposure. However, there was no detectable boost in NT50 titers following secondary, homologous DENV-1 exposures by either modality, and GMT even waned by 4- and 5-fold in the SC D1-D1 and IMF D1-D1 groups, respectively, compared to the month 13 pre-infection titers. This lack of boosting suggests negligible levels of DENV-1 replication following homologous re-exposure, which could indicate sterilizing immunity as a result of primary DENV-1 infection, irrespective of infection modality. However, the significant changes observed in hematological parameters and serum cytokine production following homologous DENV-1 exposure stand in contrast to this hypothesis and suggest a need for further characterization of fluctuations in these parameters during repeated DENV exposures, anesthetizations, and blood sample collections in this model.

### Conclusion

The present study demonstrated the effects of varying the infection modality for DENV in a nonhuman primate model, including changes to the timing and magnitude of DENV replication kinetics in the periphery and to the concentration of numerous hematological, biochemical, and immunological parameters as compared to a more typical subcutaneous inoculation. Many of these effects were serotype- or strain-dependent, and the addition of salivary gland extract to an intradermal inoculation was not sufficient to recapitulate DENV infection via IMF in the present study. DENV infection of rhesus macaques via IMF appears to be an important step forward for better recapitulating natural human infection, although additional factors could be contributing to the observed outcomes.

A potential confounding variable in the study of DENV pathogenesis following natural infection is the degree to which the subject was previously exposed to mosquito saliva and salivary antigens by blood-feeding mosquitoes. The prevalence of antibodies to the MSP of a given species in human sera varies with age and seasonality, but has been reported to be as high as 97% in *Ae*. *aegypti* endemic areas.[71–74] A significant association was also found between the presence of antibodies to individual *Ae*. *aegypti* salivary proteins in sera from patients with secondary dengue infections and their clinical presentation.[75] Additionally, pre-exposure of mice to feeding by *Ae*. *aegypti* resulted in increased WNV neurovirulence and mortality upon subsequent viral infection via vector mosquito.[56] The macaques in the present study were obtained from colonies that are housed outdoors, and all have been exposed to the bites of various species of mosquito naturally in that environment. Given the improbability of locating a cohort of MSP-naïve animals, such a control group was not included and, thus, the impact of sensitization to MSP on infection cannot be parsed out from the physiological impact of MSP on infection in a MSP-naïve system.

Further, as discussed above, the quantity of virus inoculated by a probing and feeding mosquito relative to the amount needle-inoculated in this study remains uncharacterized. While we cannot exclude that the increased peak titers seen in the mosquito-exposed animals might be due to a higher overall inocula per animal, this is unlikely in light of other published literature. A systematic review of NHP models of DENV concluded that increasing dose was significantly associated with shorter time until detectable viremia and a shorter duration of detectable viremia, whereas these times in the present study were similar or increased in the mosquito-exposed animals relative to needle-inoculated animals.[63] Additionally, another review of NHP models of DENV concluded that higher doses do not appear to result in higher viremias, including doses as high as 10^7^ PFU injected intravenously.[76]

Differences in early viral recognition and effector molecule production associated with the challenge modality chosen when assessing vaccine efficacy could have great influence over downstream replication and pathogenesis of DENV, and thereby influence the apparent efficacy of a candidate vaccine. The data presented herein further points to a need for inclusion of vector-based administration in the investigation of other arbovirus systems of human public health relevance.

## Materials and methods

### Ethics statement

Research was conducted under an animal use protocol approved by the WRAIR/NMRC Institutional Animal Care and Use Committee. An institutional approval letter for this study has been provided to the journal. Research was conducted in an AAALACi accredited facility in compliance with the Animal Welfare Act and other federal statutes and regulations relating to animals and experiments involving animals and adheres to principles stated in the *Guide for the Care and Use of Laboratory Animals*, NRC Publication, 2011 edition. Material has been reviewed by the Walter Reed Army Institute of Research. There is no objection to its presentation and/or publication. The opinions or assertions contained herein are the private views of the author, and are not to be construed as official, or as reflecting true views of the Department of the Army or the Department of Defense.

Animals were housed individually during the viremic periods of the study, and pair-housed for the remainder of the study on a 12:12-hour light cycle and with 10–15 room air changes per hour. Animals were fed Old World Primate Chow 5038 (Quality Lab Products, Elkridge, MD) twice daily, fresh fruit at least three times a week, and water *ad libitum*. Environmental Enrichment was provided in the form of cage complexities, food treats, opportunities to forage, a rotation of several toys and puzzles, periodic access to an activity cage that permits climbing, jumping, and swinging, and alternating days of television and music. Animals were anesthetized with a combination of ketamine (11 mg/Kg) and acepromazine (0.55 mg/Kg), intramuscularly, prior to all procedures. The studies performed under this protocol were nonterminal.

### Animals

Acquisition of healthy, adult, female, Chinese origin, colony bred rhesus macaques was facilitated by Covance Research Products and the NIH Primate Center. Prior to being placed on protocol all animals were tested and found negative for antibodies to Japanese encephalitis virus, WNV, yellow fever virus, and DENV-1-4 by a low-dilution screening using the virus neutralization assay prior to initial infection.

### Study design

Animals were assigned in no particular order, while attempting to balance ages and weights, to four experimental infection groups. These groups differed in virus exposure modality as shown in Table 1. Prior to infection all animals were pre-exposed to feeding *Aedes aegypti* (Rockefeller strain) mosquitoes to prime them immunologically to mosquito salivary antigens. Cartons of 20 mosquitoes were allowed to feed on each animal at 7, 5, and 4 weeks prior to infection. Four weeks after the last mosquito priming, animals were infected with DENV-1 according to group. Virus was administered into the shaved skin in separate locations spaced at least 3 cm apart at the dorsal aspect by the methods specified in Table 1. Skin punch biopsies were collected immediately after infection, and at 6h, 24h, and 21 days post-infection to characterize inflammation and transcriptional differences at the infection site. Blood samples were collected prior to infection for baseline and then after infection at 6 hours, daily from day 1 to 14, and on day 16, 18, 21, and 35 to assess circulating infectious virus (viremia) and viral RNA (RNAemia), serum antibodies, cytokines, CBC/differential, and serum chemistries. Following primary infection, follow-up blood samples were collected at approximately monthly intervals to allow for longitudinal assessment of serum neutralizing antibody titers before administering the secondary infection. Weight, body temperature, pulse and respiration rates were recorded for each animal at every time point. After assessment of primary infection, the SC and IMF groups were selected for secondary infection administered approximately 15 months after the primary infection. The 10 animals in each of the original SC and IMF groups were divided into subgroups of 5 animals each. Two new flavivirus-naïve control groups of 5 animals each (SC N-D2 and IMF N-D2) were added to the protocol to serve as primary DENV-2 infection controls as shown in Table 1, and pre-exposed to feeding *Ae*. *aegypti* as above. The animals then were exposed by group to either DENV-1 (homologous re-exposure) or DENV-2 (heterologous exposure or primary exposure in the case of the flavivirus-naïve controls) and followed as in primary DENV-1 infection. For both study phases, animals were monitored for the development of clinical signs of disease, and blood samples and infection site skin biopsies were collected for assessment of virological, hematological, and immunological parameters.

### DENV administration methods

Animals were exposed to virus by one of four methods: (i) subcutaneous injection (SC), (ii) intradermal injection (ID), (iii) intradermal injection also containing mosquito salivary gland extract (ID+SGE), and (iv) feeding by infectious mosquitoes (IMF). For all injections, a DENV stock in Eagle’s Minimal Essential Medium with 20% heat-inactivated fetal bovine serum (EMEM/20% FBS) was further diluted, as needed, in un-supplemented EMEM to obtain a viral load in the final inoculum of 5.18 log_10_ PFU per animal, with administration at three separate sites of the dorsal aspect of the torso. With the exception of mosquito feeding and unless otherwise noted, the same viral dose was administered at each injection site. In four animals, a partial dose was delivered in the ID+SGE group due to clogging of the microneedle in the delivery apparatus; the dose delivered ranged from 50–90% of the intended volume, which equates to 4.88–5.13 log_10_ PFU and was considered negligible. SC injections utilized 0.5 mL doses and 1 mL syringes with 25 gauge, ¾-inch needles. ID injections utilized 120 μL doses and were administered using the Debioject device (Debiotech SA, Switzerland) equipped with a microneedle. ID+SGE injections each additionally included the equivalent of five salivary glands in PBS (to equate, at a minimum, the feeding of five mosquitoes). SGE was prepared as previously described and was 0.2μm filtered prior to use.[77] For the IMF groups, 5 mosquitoes in a canister with a 10mm restricted opening were allowed to feed per each of three sites, totaling 15 mosquitoes per animal. The mosquitoes were allowed to feed until 5 had probed and at least 3 fed to repletion per location or up to ten minutes (S4 Table).

### Cells and viruses

The DENVs used for NHP infection were recently circulating strains from Brazil isolated in C6/36 mosquito cells from febrile patients from Rio de Janeiro (DENV-1 strain 0111/2011) and Campos (DENV-2 strain 0126/2010) in 2011 and 2010, respectively. These viruses were kindly provided by Dr. Ana Bispo de Filippis and described previously.[22] To prepare stocks for NHP infection, Vero cell culture supernatants containing 20% FBS were harvested and centrifuged to pellet large debris, and clarified supernatants then were plaque titrated. Single-use aliquots were frozen at -80°C prior to use. Vero passage-2 stocks, following the original isolation in C6/36 cells, were used for infection of NHP and mosquitoes. The stocks were sequenced using DENV1 and DENV2 specific primers, respectively, on the Illumina MiSeq platform as described in [78] to authenticate contents. The genomes were assembled using an in-house developed reference mapping pipeline, ngs_mapper (https://github.com/VDBWRAIR/ngs_mapper).

Additionally, DENV1-4 (strains Western Pacific 1974, S16803, CH53489, TVP-360, respectively), YFV strain 17D, and JEV strain SA14-14-2 were utilized for ELISA, FlowNT50, and/or PRNT assays. Viruses for PRNT assays were produced in Vero cells. Viruses for ELISA and FlowNT50 assay were produced in C6/36 cells. Virus for ELISA was purified by ultracentrifugation through a 30% sucrose solution, and the virus pellet was resuspended in PBS.

### Infection of mosquitoes

Laboratory strain *Ae*. *aegypti* Rockefeller were maintained under constant environmental conditions (28–29°C, 60–80% relative humidity, with a 12:12-hour light:dark photoperiod) and provided 10% sucrose solution ad libitum. Mosquitoes were cold-anesthetized and then exposed to DENV by intrathoracic (IT) inoculation using a microcapillary needle fixed to a microinjection apparatus. Mosquitoes were injected with 0.3μL of 7 log_10_ PFU/mL DENV1 or 6.75 log_10_ PFU/mL DENV2 stocks. Mosquitoes were utilized for feeding on NHP on day 14 post IT inoculation. Method validation prior to study start demonstrated 100% infection of mosquito salivary glands by either stock virus as measured using plaque titration.

### Biopsy collection

Following mosquito feeding or needle inoculation, the sites of administration were biopsied in time course sequence using 10mm biopsy punches. These skin biopsies were then dissected into cross-sectional portions. One portion was immediately submerged in 10% neutral buffered formalin for histopathological analysis. In the case of primary DENV-1 infection, another portion was immediately submerged in RNA-later solution (Thermo Fisher Scientific), according to manufacturer’s suggested volume and preservation time, for subsequent RNA extraction. The third virus administration site was not biopsied until after the systemic infection had cleared, on day 21.

### PRNT

Standard plaque-reduction neutralization tests (PRNT) on Vero cell monolayers were used to screen pre-study sera for determining serologically flavivirus-naïve animals at a 1:10 dilution of heat-inactivated sera.

### FlowNT50

Neutralizing antibody titers in heat-inactivated sera between the primary infection and secondary infection phases of the experiment were determined using a 96-well, high-throughput, flow cytometry-based neutralization assay as previously described.[79] Serial dilutions of sera were mixed with an equal volume of virus, diluted to achieve 10–15% infection of cells/well, and incubated for 1h at 37°C. After 1h of incubation, an equal volume of medium (RPMI-1640 supplemented with 10% FBS, 1% penicillin/streptomycin, 1% L-glutamine (200mM), and 1% nonessential amino acids (10mM)) containing 5x10^4^ U937-DC-SIGN cells were added to each serum-virus mixture and incubated 18–20 h overnight in a 37°C, 5% CO2, humidified incubator. Following overnight incubation, the cells were fixed, permeabilized and immunostained with the flavivirus group-reactive mouse monoclonal antibody 4G2, and a secondary polyclonal goat anti-mouse IgG PE-conjugated antibody (#550589, BD Biosciences). The percent infected cells were quantified on a BD Accuri C6 Plus flow cytometer (BD Biosciences). Data were analyzed by nonlinear regression to determine 50% endpoint titers in GraphPad Prism 6.

### Antibody-dependent enhancement assay

In vitro antibody-dependent enhancement of DENV infection was quantified as previously described.[79] Beginning at 1:40, two-fold serial dilutions of heat-inactivated day 0 sera were incubated with virus (in sufficient amount to infect 10–15% of U937-DC-SIGN cells) at 1:1 for 1h at 37°C. This mixture was then added to a 96-well plate containing 5x10^4^ cells (K562) per well in duplicate. Cells were infected 18–20 h overnight in a 37°C, 5% CO2, humidified incubator. Processing and quantification continued as outlined in the FlowNT50 methods. Fold-infection relative to control serum was reported.

### Plaque titration

Plaque titration of DENV in inocula and in sera were determined by standard plaque assay, in duplicate, on Vero cell monolayers. Limit of detection was 25 plaque-forming units/mL (PFU/mL) for primary infection samples and 5 PFU/mL for secondary infection samples. Values for undetectable samples were replaced with 12.5 or 2.5 PFU/mL, respectively, for the purposes of statistical analysis and graphing; these values are one-half of the theoretical minimum of one detected plaque multiplied by the assay dilution factor of 25 or 5, respectively, to achieve per mL concentrations.

### Virus quantification (qRT-PCR)

DENV in sera was determined as previously described, with modification: the DENV-2 probe fluorophore was changed to JOE.[80] RNA was extracted from sera on days 1–14, 16, 18, and 21 using QIAamp Viral RNA mini on the QIAcube instrument. Eluted RNA was quantified by qRT-PCR using the SuperScript III Platinum One-Step qRT-PCR kit (Thermo Fisher Scientific) on the QuantStudio 7-Flex Real-Time PCR instrument (Thermo Fisher Scientific). DENV RNAemia was calculated as genome equivalents (GE) per mL using an internal standard curve of 10-fold serially diluted in vitro-transcribed RNA. The limit of quantitation, defined here as detection of a standard curve dilution in ≥95% of at least 20 replicate curves tested, for both the DENV-1 and DENV-2 assays is 50 GE/reaction. Values for undetectable samples were replaced with 25 GE/mL for the purposes of statistical analysis and graphing; this value is one-half of a theoretical minimum of one detected copy per reaction multiplied by the assay dilution factor of 50 to achieve per mL concentrations.

### Cytokine analysis

The MILLIPLEX MAP Non-Human Primate Cytokine/Chemokine Panel I and II kits (EMD Millipore) were used to determine the concentrations of 25 different cytokines, chemokines, and growth factors present in serum: Eotaxin, IP-10/CXCL10, G-CSF, GM-CSF, IFN-γ, IL-1RA, IL-1β, IL-2, IL-4, IL-5, IL-6, IL-8, IL-10, IL-12/23 (p40), IL-13, IL-15, IL-17, IL-18, MCP-1, MIP-1α, MIP-1β, CD40L, TGF-α, TNF-α, and VEGF. The kits were used according to the manufacturer’s instructions. The data were acquired on a MAGPIX instrument (Bio-Rad) and processed using xPONENT software (Luminex Corp.). Protein standards were provided in the kit, and standard curves were generated with 7 or 6 standard dilutions (panel I—undiluted, 1:4, 1:16, 1:64, 1:256, 1:1024, 1:4096; panel II—undiluted, 1:4, 1:16, 1:64, 1:256, 1:1024) in Assay Buffer. Concentrations below the range of the standard curve were replaced with half of the minimum curve value. Concentrations beyond the range of the standard curve were replaced with the maximum curve value.

### Transcriptional analysis (RT-qPCR)

Skin biopsy cross-sectional portions were disrupted and homogenized in 750 μL of Lysis/Binding Buffer (Thermo Fisher Scientific) using the FastPrep-24 homogenizer and Lysing Matrix Z (MP Biomedicals) in accordance with the manufacturer's instructions. RNA was then purified with the *mir*Vana miRNA Isolation Kit (Thermo Fisher Scientific) in accordance with the manufacturer's instructions. Fifty μL of eluate was DNase-treated with TURBO DNA-free kit (Thermo Fisher Scientific) in accordance with the manufacturer’s Rigorous DNase treatment procedure. RNA in the resulting DNase-treated sample was further purified using the RNA Clean & Concentrator-5 kit (Zymo Research) in accordance with the manufacturer's instructions. A uniform quantity of total RNA per skin biopsy, as determined by NanoDrop ND-1000 (Thermo Fisher Scientific) A260 reading, was used to create cDNA. cDNA creation was performed with the SuperScript VILO cDNA Synthesis Kit using 500ng total RNA. Transcript quantification was performed using PowerUp SYBR Green Master Mix (Thermo Fisher Scientific) according to manufacturer’s instructions and cycling parameters on the QuantStudio 7 Flex Real-Time PCR System in triplicate in 384-well format (Thermo Fisher Scientific). Testing was conducted with 500nM of individual primer sets (Integrated DNA Technologies) for Eotaxin, IFN-β1, IL-1β, IL-4, IL-5, IL-8, IL-10, IL-29-like/IFNλ1, IP10/CXCL10, RelA, TLR3, TLR4, TLR7, TNF, IRF3, RANTES, XRN1, MDA5, RIG-I, MYD88, ALG9, and RPL32-like (S1 Table). The ΔΔCt method was used in order to calculate fold change differences for each sample at each time point relative to time 0.[44, 81] Ct values from triplicate wells were averaged to find mean-Ct values for each gene of interest per sample. Mean-Ct values for ALG9 and RPL32-like were averaged together to generate the mean normalization factor, which was then utilized as the “house-keeping gene” in ΔCt calculations. Undetectable values were artificially set to the maximum detected Ct value per target rounded up to the nearest 0.5Ct in order to enable relative quantification.

### Histopathology

Histopathological analysis was conducted on all skin biopsy specimens. Tissue sections were immersion-fixed in 10% neutral buffered formalin, embedded in paraffin, sectioned, and stained with hematoxylin and eosin. Dermatitis/panniculitis was scored from 0–4 (none, minimal, mild, moderate, marked) by board-certified anatomical pathologists.

### Clinical chemistry and hematology

Hematologic analysis was performed using whole blood samples collected in purple-topped EDTA tubes on days 0, 7, and 21. Hematologic analysis was conducted using a Sysmex XT-2000iV Hematology Analyzer (Sysmex America, Lincolnshire, IL). The hematology parameters analyzed included white blood cell (WBC) count, red blood cell (RBC) count, hemoglobin (HGB), percentage hematocrit (HCT), mean corpuscular volume (MCV), mean corpuscular hemoglobin (MCH), mean cell hemoglobin concentration (MCHC), platelet (PLT) count, red cell distribution width (RDW), mean platelet volume (MPV), reticulocyte percentage, and reticulocyte count. Reference ranges utilized were in-house intervals based upon routine physicals of 200 rhesus monkeys from the WRAIR animal colony not involved in studies. Serum chemistry analysis was obtained from whole blood collected in gold-topped serum separator tubes. Serum was analyzed for aspartate aminotransferase (AST) and alanine transaminase (ALT) using a Vitros 350 Chemistry System (Ortho Clinical Diagnostics, Raritan, NJ).

### Statistical analyses

Statistical analyses were performed according to the data distribution and type as follows: 1) RNAemia and viremia data were compared in the following ways: AUC, peak magnitude, duration, and onset. AUC and peak magnitude were analyzed using log-transformed values while onset and duration were analyzed using untransformed values. Differences were tested for using ANOVA and estimates obtained. For AUC and peak magnitude, these estimates are means of log-values which are geometric means. For results presentation, the ratios of the geometric means were calculated for each pairwise comparison and are discussed as fold changes. For the onset and duration, estimates obtained are differences in means and presented as such. 2) Statistical significance of clinical values was assessed as the change from baseline and analyzed via ANOVA, with the baseline values of the variable, baseline weight, and group as factors. 3) Analyses of transcriptional data were based on fold change from baseline derived from the ΔΔCt method; fold change values between 0.5–2 were considered not biologically meaningful. 4) For both transcriptional data and antibody data, to compare each group to the SC group, an ANOVA model was fitted on fold changes of log_2_ (transcriptional data) or log_10_ (antibody data) by including group, time, and group*time as fixed effects. Time was considered as a repeated measure with an unstructured covariance matrix, and heterogeneous variances between groups were assumed. Geometric means and geometric mean ratios and their respective 95% CIs were derived from this model. No adjustment for multiplicity was performed. All data analyses were performed using SAS version 9.4 or RStudio with R version 3.5.1. All tests performed as two-tailed tests and significance was assessed at α = 0.05.

## Supporting information

S1 Fig Hematology and clinical chemistries following primary and secondary DENV exposures. Data were measured on study days 0, 7, and 21 post A) primary DENV-1 infection and B) homologous DENV-1 re-exposure, heterologous DENV-2 infection, and primary DENV-2 infection. n = 10 for each SC, ID+SGE, and IMF; n = 9 for ID; n = 5 for the remainder of groups. (*, *p*<0.05). SC, subcutaneous; ID, intradermal; ID+SGE, intradermal + salivary gland extract; IMF, infectious mosquito feeding; D1-D1, homologous DENV-1; D1-D2, heterologous DENV-2; N-D2, primary DENV-2.
(TIF)

S2 Fig Dermatitis/panniculitis scores for DENV administration site skin biopsy specimens. The number of animals with dermatitis/panniculitis scores of 1 (minimal) or 2 (mild) in their administration site skin biopsy are shown by group and time point following A) primary DENV-1 infection and B) homologous DENV-1 re-exposure, heterologous DENV-2 infection, and primary DENV-2 infection. n = 10 for each SC, ID+SGE, and IMF; n = 9 for ID; n = 5 for the remainder of groups. SC, subcutaneous; ID, intradermal; ID+SGE, intradermal + salivary gland extract; IMF, infectious mosquito feeding; D1-D2, heterologous DENV-2; N-D2, primary DENV-2.
(TIF)

S3 Fig Geometric means of fold change from baseline by day of serum cytokines following primary and secondary DENV exposures. Serum was collected at 0 hour, 6 hours, and days 1, 3, 5, 7, 10, and 16 A) post-primary DENV-1 infection, B) post-primary and post-secondary DENV-1 for SC and IMF animals only, and C) post-primary and post-secondary DENV-2 infection. Twenty-five cytokines, chemokines, and growth factors in serum were assessed. Data are expressed as geometric mean fold change ranges relative to baseline (0h). Fold change values within 2-fold of baseline (horizontal grey bands) were considered unchanged from baseline. n = 10 for each SC, ID+SGE, and IMF; n = 9 for ID; n = 5 for the remainder of groups. SC, subcutaneous; ID, intradermal; ID+SGE, intradermal + salivary gland extract; IMF, infectious mosquito feeding; D1-D1, homologous DENV-1; D1-D2, heterologous DENV-2; N-D2, primary DENV-2.
(TIF)

S4 Fig Longitudinal neutralizing antibody titers for individual animals following primary DENV-1 infection. NT50 titers for months 1, 5, 8, 10, and 13 post infection sera against the four DENV serotypes are shown from the A) subcutaneous (SC) and B) infectious mosquito feeding (IMF) groups.
(TIF)

S5 Fig Antibody-dependent infection enhancement curves for individual animals following primary DENV-1 infection. Fold increase in infection of K562 cells with DENV-2 in the presence of serial dilutions of sera for individual animals from the A) SC and B) IMF groups. The corresponding NT50 titer for each animal is shown as a red dotted line.
(TIF)

S1 Table Transcriptional analysis primer sets. (XLSX)

S2 Table Table of additional hematology and clinical chemistry significant differences. (XLSX)

S3 Table Neutralizing antibody titers before and after homologous DENV-1, heterologous DENV-2, and primary DENV-2 exposure. NT50 titers on day 35 post exposure are shown for each animal, as well as geometric mean titers (GMT) for each group. Fold change values for each animal and geometric mean fold change values for each group relative to pre-secondary exposure titers (month 13) are also shown.
(XLSX)

S4 Table Table of counts of infectious mosquito feeding for individual animals. The number of mosquitoes that fed per each of the three exposure locations on the back of each animal, as well as the total number of fed mosquitoes per animal is shown. Counts are provided for both primary (IMF) and secondary (IMF D1-D1, IMF D1-D2, and IMF N-D2) exposures.
(XLSX)
