# Supplementary figures and images for: Route of inoculation and mosquito vector exposure modulate dengue virus replication kinetics and immune responses in rhesus macaques
Source: PLoS Negl Trop Dis. 2020 Apr 8;14(4):e0008191. doi: 10.1371/journal.pntd.0008191 (PMC7141610; doi:10.1371/journal.pntd.0008191)

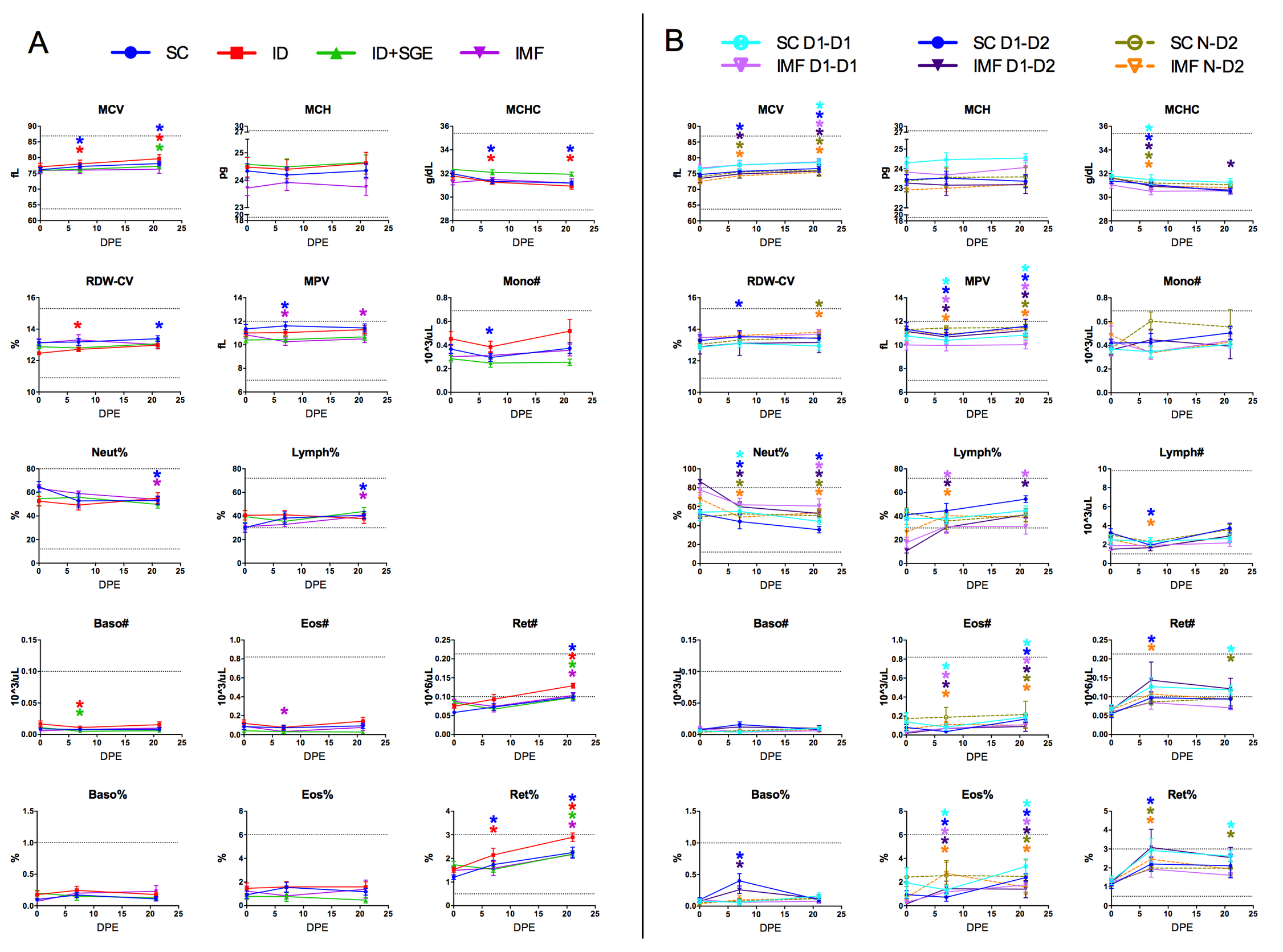

Supplement: S1 Fig — Data were measured on study days 0, 7, and 21 post A) primary DENV-1 infection and B) homologous DENV-1 re-exposure, heterologous DENV-2 infection, and primary DENV-2 infection. n = 10 for each SC, ID+SGE, and IMF; n = 9 for ID; n = 5 for the remainder of groups. (*, p<0.05). SC, subcutaneous; ID, intradermal; ID+SGE, intradermal + salivary gland extract; IMF, infectious mosquito feeding; D1-D1, homologous DENV-1; D1-D2, heterologous DENV-2; N-D2, primary DENV-2. (TIF) [file pntd.0008191.s001.tif]

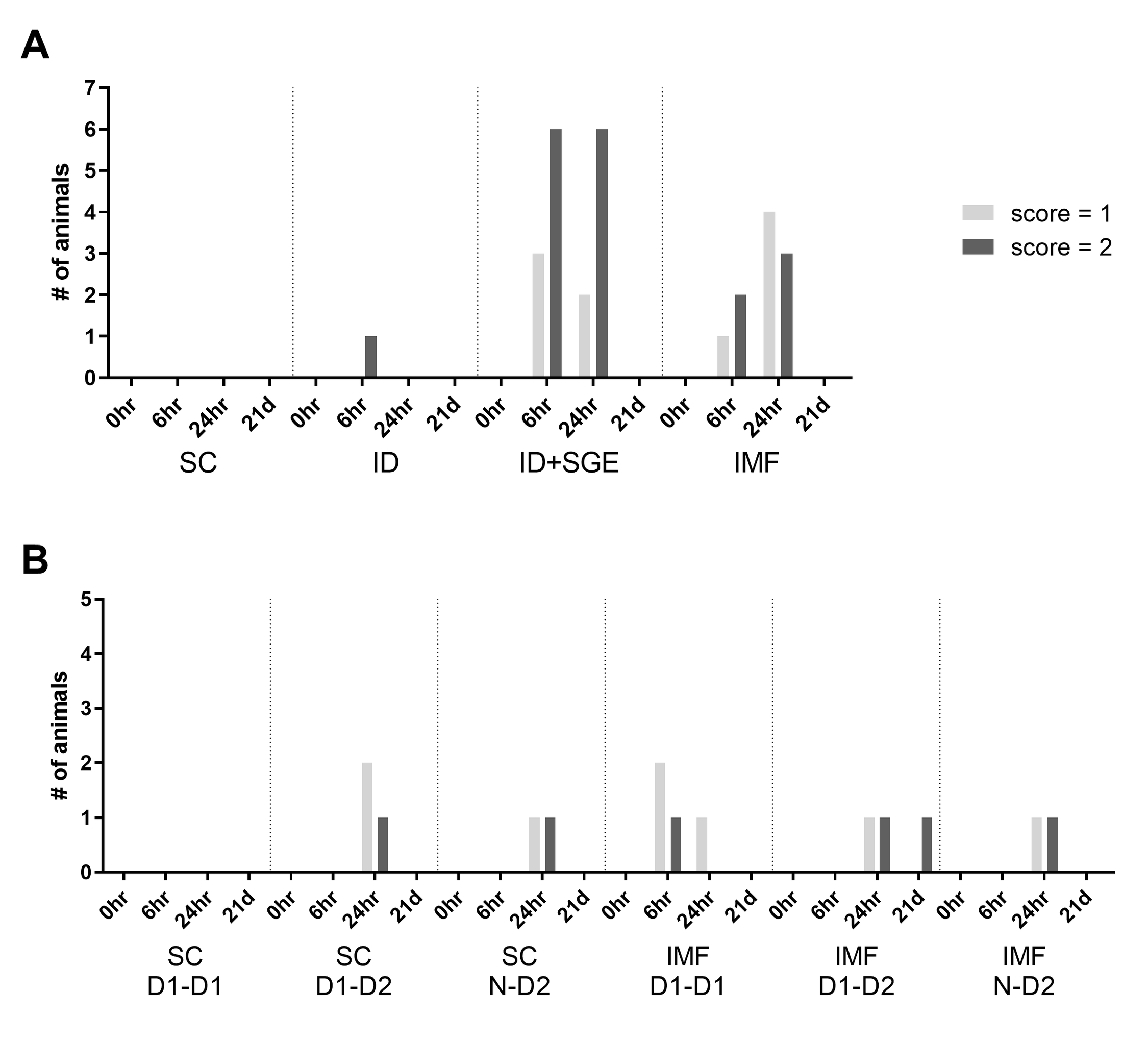

Supplement: S2 Fig — The number of animals with dermatitis/panniculitis scores of 1 (minimal) or 2 (mild) in their administration site skin biopsy are shown by group and time point following A) primary DENV-1 infection and B) homologous DENV-1 re-exposure, heterologous DENV-2 infection, and primary DENV-2 infection. n = 10 for each SC, ID+SGE, and IMF; n = 9 for ID; n = 5 for the remainder of groups. SC, subcutaneous; ID, intradermal; ID+SGE, intradermal + salivary gland extract; IMF, infectious mosquito feeding; D1-D2, heterologous DENV-2; N-D2, primary DENV-2. (TIF) [file pntd.0008191.s002.tif]

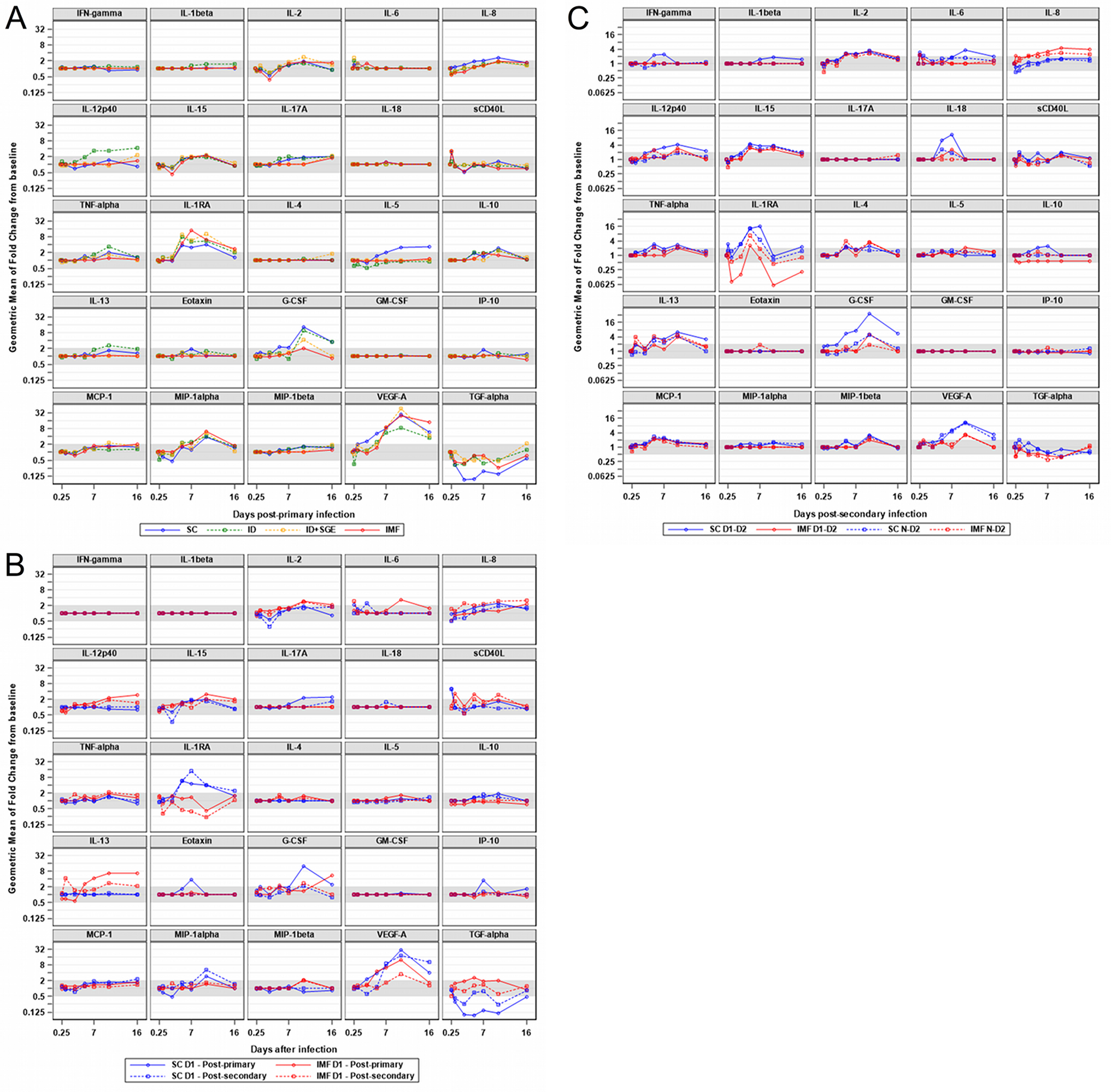

Supplement: S3 Fig — Serum was collected at 0 hour, 6 hours, and days 1, 3, 5, 7, 10, and 16 A) post-primary DENV-1 infection, B) post-primary and post-secondary DENV-1 for SC and IMF animals only, and C) post-primary and post-secondary DENV-2 infection. Twenty-five cytokines, chemokines, and growth factors in serum were assessed. Data are expressed as geometric mean fold change ranges relative to baseline (0h). Fold change values within 2-fold of baseline (horizontal grey bands) were considered unchanged from baseline. n = 10 for each SC, ID+SGE, and IMF; n = 9 for ID; n = 5 for the remainder of groups. SC, subcutaneous; ID, intradermal; ID+SGE, intradermal + salivary gland extract; IMF, infectious mosquito feeding; D1-D1, homologous DENV-1; D1-D2, heterologous DENV-2; N-D2, primary DENV-2. (TIF) [file pntd.0008191.s003.tif]

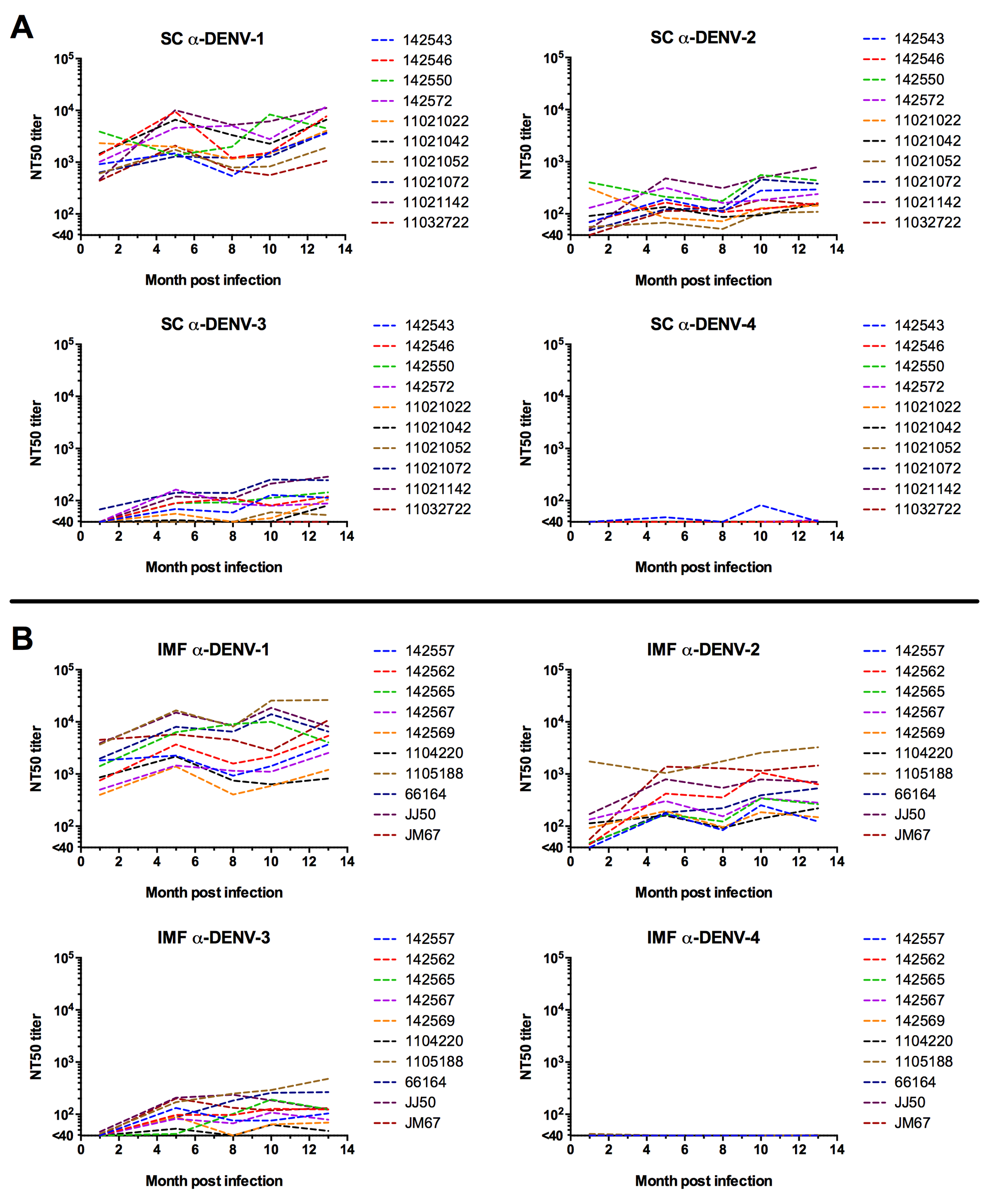

Supplement: S4 Fig — NT50 titers for months 1, 5, 8, 10, and 13 post infection sera against the four DENV serotypes are shown from the A) subcutaneous (SC) and B) infectious mosquito feeding (IMF) groups. (TIF) [file pntd.0008191.s004.tif]

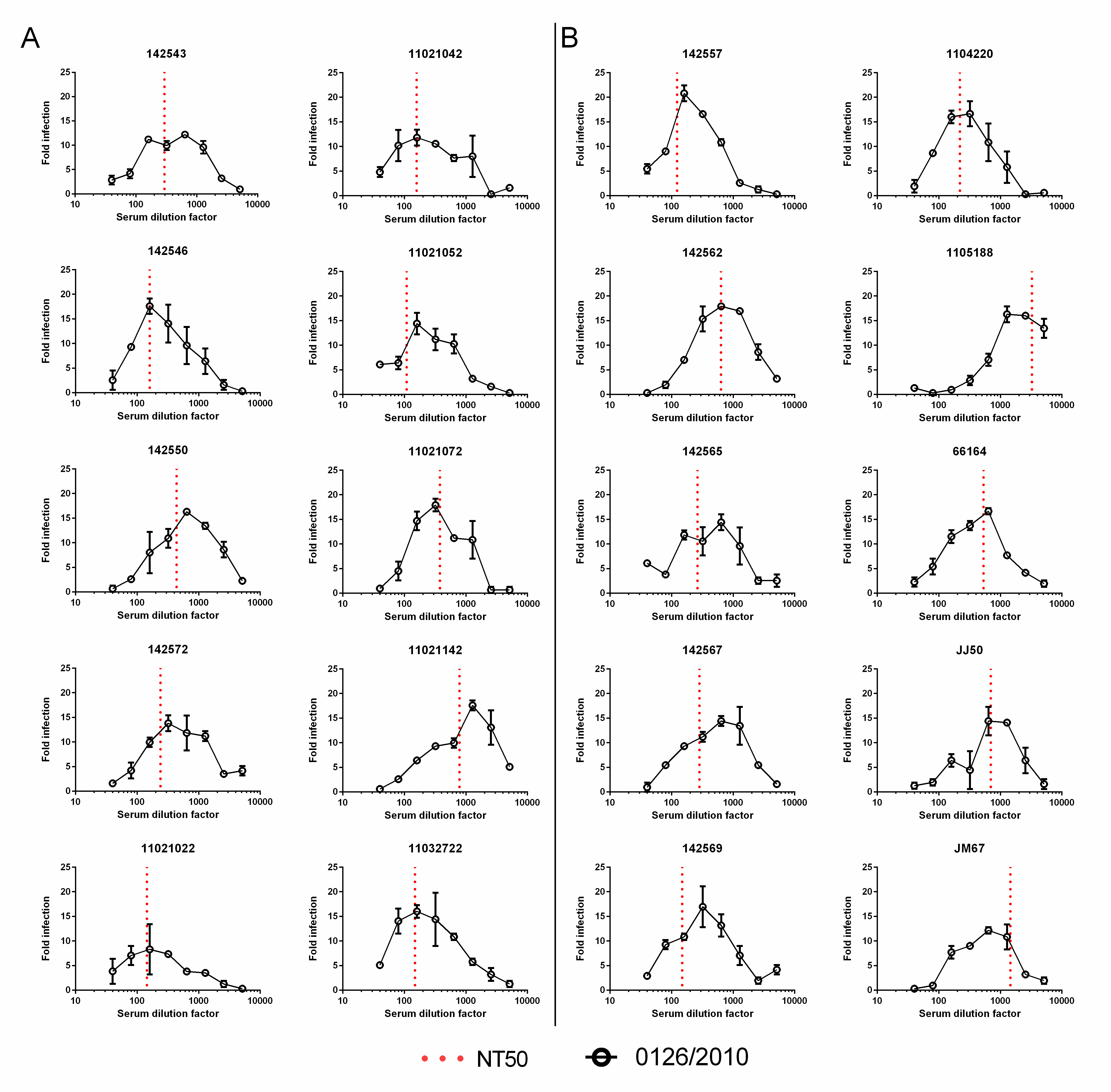

Supplement: S5 Fig — Fold increase in infection of K562 cells with DENV-2 in the presence of serial dilutions of sera for individual animals from the A) SC and B) IMF groups. The corresponding NT50 titer for each animal is shown as a red dotted line. (TIF) [file pntd.0008191.s005.tif]
